# Microneme Proteins 1 and 4 From *Toxoplasma gondii* Induce IL-10 Production by Macrophages Through TLR4 Endocytosis

**DOI:** 10.3389/fimmu.2021.655371

**Published:** 2021-04-12

**Authors:** Rafael Ricci-Azevedo, Flavia Costa Mendonça-Natividade, Ana Carolina Santana, Juliana Alcoforado Diniz, Maria Cristina Roque-Barreira

**Affiliations:** ^1^ Laboratory of Immunochemistry and Glycobiology, Department of Cell and Molecular Biology and Pathogenic Bioagents, Ribeirão Preto Medical School, University of São Paulo, Ribeirão Preto, Brazil; ^2^ Laboratory of Cellular and Molecular Biology of Mast Cells, Department of Cell and Molecular Biology and Pathogenic Bioagents, Ribeirão Preto Medical School, University of São Paulo, Ribeirão Preto, Brazil; ^3^ Laboratory of Molecular Parasitology, Department of Cell and Molecular Biology and Pathogenic Bioagents, Ribeirão Preto Medical School, University of São Paulo, Ribeirão Preto, Brazil

**Keywords:** *Toxoplasma gondii*, MIC1, MIC4, TLR4 (Toll-like receptor 4), Dynasore, endocytosis, IL-10 (Interleukin 10)

## Abstract

The protozoan parasite *Toxoplasma gondii* modulates host cell responses to favor its success in the early stage of infections by secreting proteins from its apical organelles. Some of these proteins, including microneme proteins (MICs) 1 and 4, trigger pro-inflammatory host cell responses. The lectins MIC1 and MIC4 interact with N-linked glycans on TLR2 and TLR4, activating NF-κB and producing IL-12, TNF-α, and IL-6. Interestingly, MIC1 and MIC4 also trigger secretion of the anti-inflammatory cytokine IL-10 through mechanisms as yet unknown. Herein, we show that the ability of these MICs to induce macrophages to produce IL-10 depends on TLR4 internalization from the cell surface. Macrophages subjected to blockade of endocytosis by Dynasore continued to release TNF-α, but failed to produce IL-10, in response to MIC1 or MIC4 exposure. Similarly, IL-10 was not produced by Dynasore-conditioned *T. gondii*-infected macrophages. Furthermore, MIC1- or MIC4-stimulated macrophages gained transient tolerance to LPS. We report a previously undiscovered mechanism by which well-defined *T. gondii* components inhibit a host inflammatory response.

## Introduction

*Toxoplasma gondii* is a protozoan obligate intracellular parasite of the phylum Apicomplexa, that causes toxoplasmosis. *T. gondii* infects a range of warm-blooded animals, including humans (1). From the estimated 1/3 of the world’s population chronically infected with *T. gondii*, most of them are clinically asymptomatic. There are relevant exceptions. Reactivation of latent disease in immunocompromised patients frequently causes life-threatening encephalitis, and acute infection acquired during pregnancy can be fatal to the fetus (2–4). *T. gondii* invades host cells *via* several mechanisms (5, 6), including recognition of carbohydrates on a host cell surface (7, 8). The host cell response to contact with the parasite plays a crucial role in deciding infection outcome (9).

Studies of *T. gondii* components capable of inducing cytokine production by innate immune cells have made progress in recent years. Most reports have focused on the role of profilin in activating TLR11 (10) and TLR12 (11). This activation results in release of the pro-inflammatory cytokine IL-12 (12). The ability to induce IL-12 secretion *via* TLR activation has been attributed to other *T. gondii* components, including glycosylphosphatidylinositol (GPI) anchors (13) and heat shock protein 70 (TgHSP70) (14). Granule dense proteins (GRA) 24 account for cytokine release by macrophages, which occurs through a TLR-independent pathway (15, 16). Finally, our laboratory has shown that the complex LAC+, containing the microneme proteins (MICs) 1, 4, and 6 from *T. gondii* induce cytokine release by innate immune cells (17, 18), which was later confirmed to be happening due to the interaction of MIC1 and MIC4 with both TLR2 and TLR4 (19, 20).

We and others have previously reported that MIC1 and MIC4 possess lectin domains (17, 21, 22) that recognize oligosaccharides with terminal α (2, 3)-sialyl residues linked to β-galactosides (MIC1) (17, 19) or terminal β(1–4)- or β(1–3)-galactose (MIC4) (19, 23). These carbohydrate recognition domains (CRDs) account for the interaction of MICs with glycans that are N-linked to receptors, such as TLR2 and TLR4, on innate immune cells (24). The interactions of isolated MIC1 or MIC4 with TLR2 are sufficient to trigger pro-inflammatory cytokine production. This response is optimized in the presence of the co-receptor CD14, or upon TLR2 heterodimerization with TLR1 or TLR6 (20). Remarkably, MIC1 and MIC4 also induce production of the anti-inflammatory cytokine IL-10 in addition to release of pro-inflammatory cytokines, including IL-12, IL-6, and TNF-α (19).

Following *T. gondii* infection, the IL-12 produced by mononuclear phagocytes stimulates release of IFN-γ by NK and CD4+ T cells, driving the host immune response toward a Th1 axis (12, 25, 26). Although beneficial, an exaggerated Th1 response intensifies inflammation, potentiating tissue injury unless increased IL-10 release regulates this response (27, 28). The key role of IL-10 in *T. gondii* infection was demonstrated by inoculating an avirulent parasite strain in IL-10 knock-out (KO) mice, which yielded 100% mortality within the first two weeks, although the level of parasite proliferation was similar to that detected in WT mice, which survived the infection. Compared to controls, IL-10 KO mice had four- to six-fold higher serum levels of IL-12 and IFN-γ, and their death was attributed to enhanced liver pathology, consisting of intense inflammatory cell infiltration and necrosis (29).

Some mechanisms by which MIC1 and MIC4 prime innate immune cells have been elucidated, including structural requirements and signaling cascades underlying TLR2 activation (20), but many details of cell-priming remain unknown. This study characterizes TLR4 dependent IL-10 production by MIC1- or MIC4-stimulated macrophages. MIC1/TLR4 or MIC4/TLR4 complex formation on the cell surface is sufficient to stimulate inflammatory cytokines. However, these complexes must undergo endosomal internalization to induce production of the anti-inflammatory cytokine IL-10, which can reproducibly confer cell tolerance to a subsequent inflammatory stimulus in *T. gondii*-infected macrophages. We provide the first report of a mechanism underlying production of anti-inflammatory cytokines in response to *T. gondii* proteins.

## Materials and Methods

### Animals and Ethics

All experiments were performed in accordance with the ethical principles in animal research described by the Brazilian Society of Laboratory Animal Science, and were approved by the Ethics Committee on Animal Experimentation and Research of the Ribeirão Preto Medical School (FMRP), University of São Paulo (USP) (protocol number 191/2017). C57BL/6 mice, Wild type (WT) or genetically lacking CD14 (CD14-/-), TLR2 (TLR2-/-), or TLR4 (TLR4-/-) genes, 8 to 12 weeks old, were obtained from the Central Animal Facility of the University of São Paulo in Ribeirão Preto and housed in the bioterium of the Department of Cellular and Molecular Biology – FMRP.

### MIC1 and MIC4 Recombinant Proteins

The procedures for obtaining highly purified and endotoxin-free recombinant lectins were made accordingly to the protocol previously published by us (30). Briefly, for the recombinant proteins expression, *mic1* and *mic4* sequences were amplified from cDNA of the *T. gondii* strain ME49 with a 6—histidine-tagged added on the N-terminal, cloned into pDEST17 vector (Gateway Cloning, Thermo Fisher Scientific, Grand Island, NY) under T7 promoter inducible by isopropyl-β-D-1-thiogalactopyranoside (IPTG) (Sigma Aldrich, St. Louis, MO). The MIC1 and MIC4 were then purified from inclusion bodies and refolded by gradient dialysis. The concentrations of recombinant proteins were determined by bicinchoninic acid assay (BCA) (Pierce, Thermo Fisher Scientific Inc.) and stored at –20°C. Endotoxin was removed by passing through polymyxin-B columns (Affi-Prep Polymyxin Resin; Bio-Rad, Hercules, CA) and any residual concentration were measured in all protein samples for quality control, using the Limulus Amebocyte Lysate Kit–QCL-1000 (Lonza, Basel). MIC1 and MIC4 were only used when the concentration of endotoxin was less than 3 UE/ug. Additionally, prior their use to all *in vitro* assays aliquots of the recombinant proteins were incubated with 50 µg/mL polymyxin B sulfate salt (Sigma-Aldrich) for 30 min at 37°C to neutralize any residual endotoxin. Biotinylation of MIC1 and MIC4 with Sulfo-NHS-LC-biotin (Pierce, Thermo Fisher Inc.) was performed according to the manufacturer’s recommendations kit.

### *Toxoplasma gondii* Culture

*Toxoplasma gondii* was cultured as previously described (31). Briefly, type I RH strain parasites were maintained on human foreskin fibroblast (HFF-1) monolayers, grown in Dulbecco’s modified Eagle’s medium (DMEM) supplemented with 10% (vol/vol) fetal bovine serum (FBS), 0.25 mM gentamicin, 10 U/mL penicillin, and 10 μg/mL streptomycin (Gibco, Thermo Fisher Scientific Inc.). For *in vitro* infections, parasites were recovered from HFF monolayers as previously described (19). In brief, cells were centrifuged for 5 min at 50 × g to remove HFF cell debris. Supernatant containing parasites was transferred to a new tube and centrifuged for 10 min at 1,000 × g. The pellet was resuspended in RPMI-1640 medium (Gibco,Thermo Fisher Scientific Inc.) for parasite counting.

### Macrophage Culture

WT, CD14^-/-^, TLR2^-/-^, and TLR4^-/-^ bone marrow derived macrophages (BMDMs) were obtained as previously described (32). Briefly, bone marrow cells were cultured for 7–9 days in RPMI 20/30, which consists of RPMI-1640 medium (Gibco,Thermo Fisher Scientific Inc.), supplemented with 20% (vol/vol) FBS and 30% (vol/vol) L-Cell Conditioned Media (LCCM) as a source of macrophage colony-stimulating factor (M-CSF) on non-treated Petri dishes (Optilux - Costar, Corning Inc. Corning, NY). Twenty-four hours before experiments, BMDM monolayers were detached using cold phosphate-buffered saline (PBS) (Hyclone, GE Healthcare Inc. South Logan, UT) and cultured, as specified, in RPMI-1640 (Gibco,Thermo Fisher Scientific Inc.) supplemented with 10% (vol/vol) FBS, 10 U/mL penicillin, and 10 μg/mL streptomycin, (2 mM) L-glutamine, (25 mM) HEPES, pH 7.2 (Gibco,Thermo Fisher Scientific Inc.) at 37°C in 5% CO2 for the indicated periods.

### Macrophage Endocytosis Blockage With Dynasore

For endocytosis blockage using dynasore, 5 × 10^5^ BMDMs (500 μL/well – 24 well plates) were washed with serum-free RPMI-1640 (Gibco,Thermo Fisher Scientific Inc.) and incubated for 1 hour. Then cells were cooled to 17°C and pretreated with Dynasore (80 μM, Sigma-Aldrich) for 30 minutes in serum-free RPMI-1640. Then cells were stimulated or infected as described below. The BMDMs were incubated at 37°C in 5% CO_2_ and, for 24 hours incubation, 40 μM of Dynasore was added to each 9 h of incubation period.

### Macrophage Stimulation and *In Vitro T. gondii* Infection

BMDMs were pre-treated or not with Dynasore as previously mentioned. 1 × 10^6^ BMDMs/mL were stimulated with MIC1 or MIC4 at 5 µg/mL. It was established a sub-optimal lectin concentration based on dose-response experiments, following previous studies of our group (19, 20). LPS (standard LPS, *E. coli* 0111: B4; Sigma-Aldrich, 500 ng/mL) and medium alone were used respectively as the positive and negative controls. For *in vitro* infection assay, after counted as previously mentioned, 3 tachyzoites per BMDM were added into wells (multiplicity of infection, MOI = 3). The plates were immediately centrifuged for 3 min at 200 × g to synchronize infection within BMDMs and incubated at 37°C in 5% CO2. After incubation, culture supernatants were collected for analyzing cytokine secretion, RNA was isolated for gene expression studies, and cell lysates were prepared for western analysis.

### TLR4 Surface Detection

To detect cell surface displayed TLR4, 1 × 10^5^ BMDMs cultured in black 96-well plates with clear bottoms were stimulated with medium only, LPS, MIC1, or MIC4 for the indicated periods, then fixed with 2% (vol/vol) paraformaldehyde (Sigma Aldrich). Wells were rinsed with phosphate buffered saline solution (PBS) pH 7 to 7.2 (Hyclone, GE Healthcare Inc.) supplemented with 1M glycine (Sigma Aldrich) and blocked using anti-mouse CD16/32 (FcBlock) for 1 h at room temperature. This was followed by incubation overnight at 4°C with 0.5 ng/mL rat anti-mouse TLR4 (Biolegend San Diego, CA - 117601), followed by five washes with 200 μL PBS. Secondary antibodies conjugated with Alexa Fluor 488 (Invitrogen, Thermo Fisher Scientific Inc - A21208) were added to the wells (1:1000) and incubated for 1 h at room temperature. Wells were washed 7 times with 200 μL PBS. TLR4 was detected in an FLx800 Fluorescence Microplate Reader (BioTek Instruments, Winooski, VT); excitation 485 nm, emission 528 nm). Results are expressed as median fluorescence intensity (MFI).

### Confocal Microscopy and Colocalization Analysis

To analyze the distribution of biotinylated MIC1 and MIC4 after incubation with macrophages, 2 × 10^4^ BMDMs were cultured on 13 mm diameter glass coverslips placed in 24-well plates for 18 h at 37°C in 5% CO_2_. Cells were then stimulated with 5 µg/mL biotinylated MIC1 or MIC4 and immediately incubated for 5, 10, or 15 min at 37°C in 5% CO_2_. After incubation, cells were fixed for 20 min in 2% (vol/vol) paraformaldehyde (Sigma-Aldrich) in PBS (Hyclone, GE Healthcare Inc.), washed twice with PBS, and incubated with 0.1 M glycine (in PBS) for 10 min. Cells were then permeabilized with 0.01% (vol/vol) saponin (Sigma-Aldrich) in PBS for 20 min and blocked with 7 µg/mL polyclonal donkey anti-mouse IgG (Jackson Immuno Research, West Grove, PA, 715-007-003) plus 1% (vol/vol) bovine serum albumin (BSA) (Sigma Aldrich) in PBS for 45 min. Coverslips were washed with PBS and incubated for 1 h with a mixture of mouse monoclonal IgG2b anti- TLR4 (Abcam ab22048, 1:500) and rabbit polyclonal IgG anti- EEA1 (Abcam – ab2900, 1:500), (diluted in PBS containing 1% BSA). Cells were then washed 5 X 5 min in PBS, and then incubated with a mixture of Goat anti-mouse IgG2b conjugated with Alexa Fluor 647 (Invitrogen, Thermo Fisher Scientific Inc - A-21242, 1:1000), Goat anti-rabbit IgG conjugated with Alexa Fluor 488 (Invitrogen, Thermo Fisher Scientific Inc, A-11008, 1:1000) as secondary antibodies and, Alexa Fluor 594 conjugated streptavidin (Invitrogen, Thermo Fisher Scientific Inc, S32356, 1:1000). Finally, cells were washed 10 X 5 min in PBS, rinsed quickly with ultrapure water (Milli-Q, Merck Millipore, Watford WD), and mounted with Fluoromount G (Electron Microscopy Sciences, Hatfield, PA). Cells incubated without primary antibody were used as controls, and were all negative. Cells were analyzed using a conventional Olympus BX50 fluorescence microscope (Olympus, Waltham, MA), and a Leica TCS SP5 confocal microscope (Leica Microsystems, Wetzlar). The images were obtained by a sequential acquisition of the three fluorophores to avoid crosstalk/overlap. Colocalization studies were performed in serial cuts (Z axis) of 0.17 μm each, followed by calculation of Manders coefficients. Coefficients of colocalization tM1/tM2 were calculated using FIJI software (33) and the Colocalization Threshold Plug-in developed by Tony Collins (Wright Cell Imaging Facility, Toronto, Canada). These coefficients vary from 0 to 1, corresponding to a lack of correlation and a perfect correlation, respectively. tM1 is the number of pixels (above the background) in the green channel that overlaps the pixels (above the background) in the red channel. tM2 is the number of pixels (above the background) in the red channel that overlaps the pixels (above the background) in the green channel. For immunostaining analysis of MIC1 and MIC4, the red channel was used, and for the TLR4 and EEA-1 markers, the green channel was used. Trough LUT (Look-up Table, FIJI/ImageJ) Magenta was chosen as pseudo-color for TLR4, for better visualization.

### Western Blotting Analysis

To evaluate p38 and IRF3 phosphorylation, 1 × 10^7^ BMDMs were stimulated with MIC1, MIC4, LPS, or medium for the indicated periods of time. Cells were lysed in a buffer containing 100 mM NaCl, 20 mM Tris (pH 7.6), 10 mM EDTA (pH 8), 0.5% SDS, 1% Triton X-100, and a protease inhibitor cocktail (Sigma-Aldrich). Cells were immediately transferred into liquid nitrogen, and stored at -80°C. Laemmli sample buffer was added to lysates, and samples were boiled for 10 min. Proteins were separated by SDS-PAGE on 10% (vol/vol) polyacrylamide resolving gels and transferred to nitrocellulose membranes. The membranes were blocked for 16 h at 4°C in PBS containing 0.05% (vol/vol) de Tween-20 (Sigma Aldrich) and 3% (vol/vol) BSA (Sigma Aldrich), and were then incubated for 1 h at room temperature with primary antibodies: anti-phospho-p38 MAPK (Thr180/Tyr182, 28B10, 1:100; Cell Signaling Technology, Danvers, MA -9216), anti-p38 MAPK (1:1000; Cell Signaling-9212), anti-phospho-IRF3 (Ser396 - 4D4G; 1:1000; Cell Signaling-4947), anti-IRF3 (D83B9; 1:1000; Cell Signaling-4302), and anti-β-actin (1:1000; Santa Cruz Biotechnology Santa Cruz, CA -4778) for a loading control. The same nitrocellulose membrane was then subjected to secondary probing anti-mouse IgG-HRP (1:2000) or anti-rabbit HRP (1:2000) (Invitrogen) for 30 min. The membranes were developed using chemiluminescence (0.1M Tris-HCl pH 8.5, 2.5 mM de luminol, 0.9 mM p-coumaric acid e 1% de hydrogen peroxide solution). For stripping, the immunoblot was immersed in mild stripping buffer (​15 g glycine, 1 g SDS, 10 mL Tween 20 in 1000 mL distilled water. pH 2.2), incubated at room temperature for 10 min and the immunoblot was repeat as described. The chemiluminescence detection was performed using ChemiDoc Imaging Systems (Bio-Rad Laboratories).

### ELISA

TNF-α, IL-10 (OptEIA set; BD Biosciences, San Jose, CA), and IFN-β (R&D Systems, Minneapolis, MN) concentrations in cell culture supernatants were determined by ELISA in accordance with the manufacturer’s instructions. Standard curves generated from serial dilution of a provided set of recombinant cytokines were used to determine the respective cytokine concentrations in the supernatant samples. Absorbance at 450 nm was measured using a Power Wave-X spectrophotometer (BioTek Instruments, Inc.).

### Quantitative Real-Time PCR

BMDMs (2 × 10^7^) were stimulated for 5 h with MIC1, MIC4, LPS, or medium only. RNA was extracted using Trizol Reagent (Invitrogen) and purified using the Direct-zol RNA MiniPrep Plus Kit (Zymo Research, Irvine, CA) according to the manufacturer’s instructions. cDNA was synthesized from 1.5 μg of RNA using SuperScript Reverse Transcriptase (Invitrogen) according to the manufacturer’s instructions. Quantitative real-time PCR was performed using Power SYBR Green (Applied Biosystems, Thermo Fisher Scientific Inc.) on a 7500 Real-Time PCR thermocycler (Applied Biosystems). Relative expression of transcripts was quantified using the ΔΔCt method, and *β-actin* was used as an endogenous control. The following primers were used for quantification: *β-actin* F: 5’-GATGCAGAAGGAGATCACAGCC-3’ and *β-actin* R: 5’-ACAATGAGGCCAGAATGGAGC-3’; *Il-10* F: 5’-GCTCTTACTGACTGGCATGAG-3’ and *Il-10* R: 5’-CGCAGCTCTAGGAGCATGTG-3’; *Cxcl10* F: 5’-TTTACCCAGTGGATGGCTAGTC-3’ and *Cxcl10* R: 5’-GCTTGACCATCATCCTGCA-3; *Tnf-α* F: 5’-GACGTGGAACTGGCAGAAGAG-3’ and *Tnf-α* R: 5’-GCCACAAGCAGGAATGAGAAG-3’; and *Ifn-β* F: 5’-GCACTGGGTGGAATGAGACT-3’ and *Ifn-β* R: 5’AGTGGAGAGCAGTTGAGGACA-3’.

### Statistical Analysis

Statistical analyses were performed by one-way ANOVA followed by Bonferroni’s post-test, or two-way ANOVA followed by Tukey’s post-test, as indicated. Analyses were performed using GraphPad Prism software version 8 (GraphPad, La Jolla, CA). Differences were considered significant when P values were <0.05.

## Results

### MIC1 and MIC4 Colocalize With Early Endosomes After Interacting With TLR4

**Figure 1:**
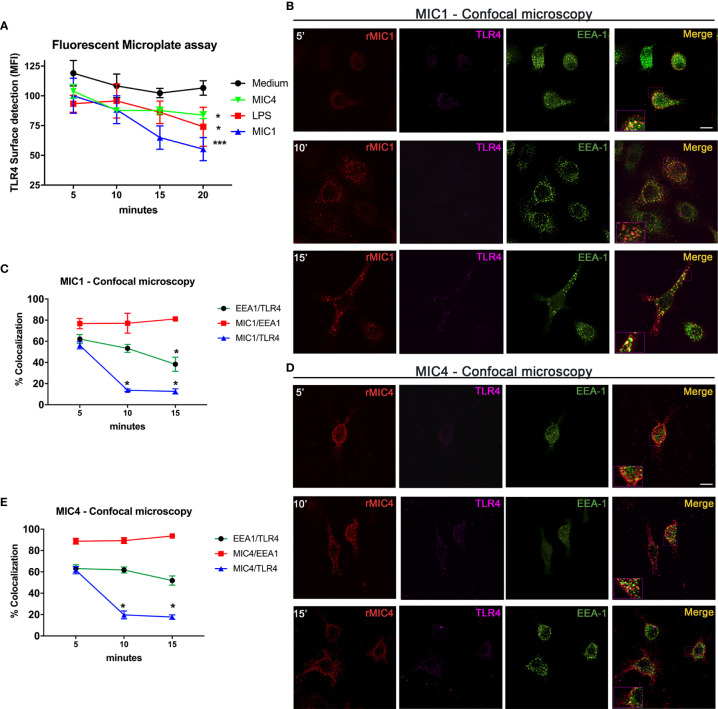
MIC1 and MIC4 colocalize with early endosomes concurrent with TLR4 downregulation on the cell surface. **(A)** BMDMs were stimulated with medium only, LPS, MIC1, or MIC4 for the indicated periods. After fixing, cells were blocked (Fcblock) and stained with anti-TLR4 antibodies. Cell surface TLR4 was quantified using an FLx800 Fluorescence Microplate Reader (BioTek Instruments, USA; excitation 485 nm, emission 528 nm). Results were expressed as MFI + SD. *p < 0.05 and *** p < 0.001 (One-way ANOVA and Bonferroni post-test). For confocal microscopy, BMDMs were incubated with biotin-MIC1 or with biotin-MIC4 for 5, 10, or 15 min **(B, D)**. After fixing and permeabilization, cells were immunostained with anti-TLR4 (Alexa-647, pseudo-color -LUT magenta), or anti-EEA-1 (Alexa-488, green). Biotinylated lectins were detected by reaction with fluorescent streptavidin (Alexa-594, red). Merge = yellow. Bar = 10 μM. Insets: 2.5-fold increased magnification. **(C, E)** Percentages of colocalization of MIC1 or MIC4 with EEA-1 (red line) or TLR4 (blue line) were determined at 5, 10, and 15 min after stimulation. Additionally, the percentage of EEA1 colocalization with TLR4 was determined (green line) for both MIC1 and MC4 stimulation at the same time points. Results were obtained by calculating the Manders Colocalization Coefficient (MCC) and expressed as averages + SD. Representative data from experiments performed in triplicate. *p < 0.05 (one-way ANOVA and Bonferroni post-test).

As previously demonstrated, *T. gondii* lectins MIC1 and MIC4 interact physically with TLR4 N-glycans on the surface of mononuclear phagocytes (19). We thus investigated the localization of the complexes following their initial contact. Using an immunofluorescence plate assay, we first detected TLR4 on the surface of vehicle control treated and MIC1, MIC4, or LPS stimulated bone marrow-derived macrophages (BMDMs). There was a significant decay in the presence of cell surface TLR4 over time, which was fastest on MIC1-stimulated cells, followed by similar rates on MIC4- and LPS-stimulated cells (**Figure 1A**).

To measure internalization of microneme protein/TLR4 complexes putatively formed on the cell surface, we performed quantitative analysis of complex component colocalization by confocal microscopy of BMDMs stimulated with biotinylated MIC1 or MIC4 for 5, 10, and 15 min. The colocalization profiles of MIC1 and MIC4 with TLR4 were similar, as shown in **Figures 1B, D**. Five minutes after stimulation, high colocalization with TLR4 was observed for MIC1 (55.2%, **Figures 1B, C**) and MIC4 (61.6% **Figures 1D, E**). These proportions decreased rapidly and significantly after longer time points: MIC1 colocalization with TLR4 was 13.7% at 10 min and 12.6% at 15 min after stimulation (**Figure 1C**). At the same time points, MIC4/TLR4 colocalization was 19.7% and 17.6% (**Figure 1E**). MIC1 and MIC4 displayed a punctate distribution throughout the cytoplasm and within various subcellular compartments, but primarily in regions cortical to the cell membrane, in a pattern that suggested endosomal encapsulation (**Figures 1B, D**). We evaluated MIC1 and MIC4 colocalization with endosomes in microneme protein-stimulated BMDMs. As expected, we did not observe colocalization of MIC1 or MIC4 with EEA1 immediately after stimulation (time zero, not shown). At 5 min, we observed approximately 80% colocalization of each MIC with EEA1. MIC1 and MIC4 maintained high-level colocalization with EEA1 for the remainder of the experimental period (**Figures 1C, E** **)**.

We next examined TLR4 colocalization with EEA-1 in MIC1- or MIC4-stimulated BMDMs. We found 60% colocalization between TLR4 and EEA-1 at 5 min post-stimulation (**Figures 1C, E** **)**. This proportion decayed significantly to 38.2% by 15 min after the MIC1-stimulus (**Figure 1C**) but was maintained at higher than 50% over time in MIC4-stimulated BMDMs (**Figure 1E**).

We conclude that microneme protein/TLR4 complexes are found early and for a brief period within stimulated cells, colocalized inside early endosomes. Although they remain within endosomes longer, MIC1 and MIC4 segregate quickly from TLR4, and are then cleared slowly and progressively from the endosome.

### TLR4 Endocytosis Is Critical for IL-10 Release From MIC1- and MIC4-Stimulated BMDMs

We have previously verified that interaction between MIC1 or MIC4 and TLR4 induces mononuclear phagocytes to release cytokines (19, 20). Herein, we showed that both MICs induce TLR4 uptake from the BMDM surface, followed by colocalization with EEA1. To evaluate the impact of TLR4 internalization on cytokine release by microneme-protein-stimulated BMDMs, we blocked their endocytic pathway with Dynasore, a dynamin inhibitor.

**Figure 2:**
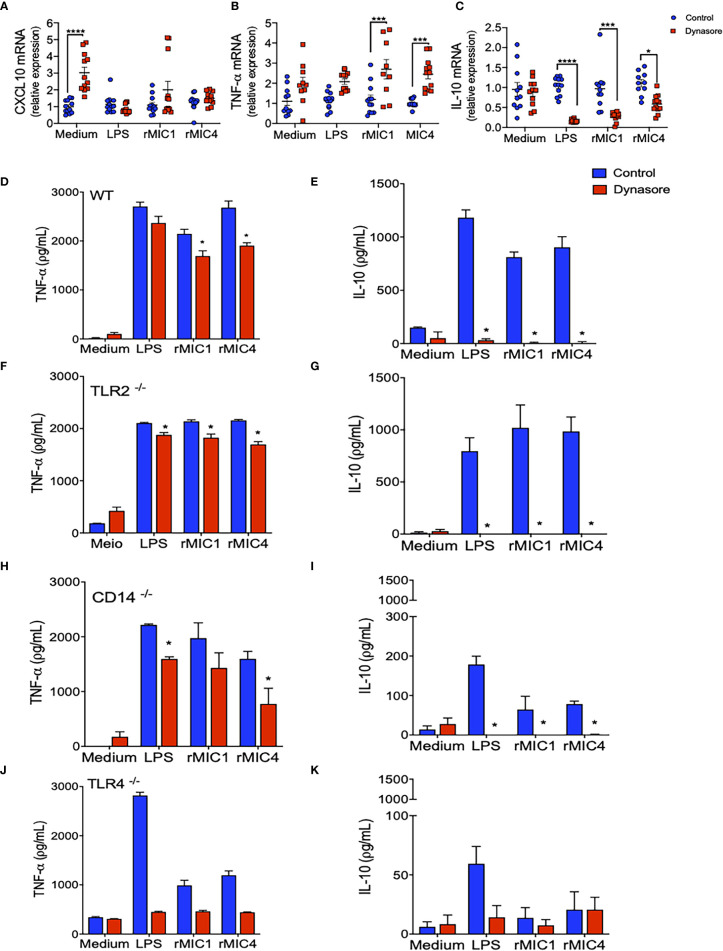
IL-10 production by MIC1- or MIC4-stimulated BMDMs requires TLR4 and a functional endocytic pathway. **(A–C)** WT BMDMs pretreated (red squares) or not (control - blue circles) with Dynasore were stimulated with Medium only, LPS, MIC1, or MIC4 for 5 h. Extracted RNA was reverse transcribed into cDNA, and expression of *Cxcl10* **(A)**, *Tnf-α* **(B)**, and *Il-10* **(C)** was analyzed by real-time PCR. Relative expression was determined as described in “Materials and Methods,” and results obtained under Dynasore treatment were compared with control conditions under the same stimulation. Results are expressed as averages + SD of four experiments, each performed in triplicate. Statistical analysis was done by two-way ANOVA followed by Tukey’s test (* = p < 0.05, *** = p < 0.001, **** = p < 0.0001). BMDMs from WT **(D, E)**, TLR2^-/-^ **(F, G)**, CD14^-/-^ **(H**, **I)**, and TLR4^-/-^ **(J, K)** mice were pretreated (red bars) or not (control - blue bars) with Dynasore and then stimulated with medium only, LPS, MIC1, or MIC4. After 24 h, cell supernatants were analyzed by ELISA for TNF-α **(D, F, H, J)** and IL-10 **(E, G, I, K)** levels. Results are expressed as averages + SD of three independent experiments, each performed in triplicate. Statistical analysis was done by two-way ANOVA followed by Tukey’s test (* = p < 0.05 in comparison with control under same stimulation).

We evaluated the impact of endocytic pathway blockade on different intracellular signaling pathways in BMDMs untreated with MICs. The impact was indirectly assessed by quantitating *Cxcl10* (dependent on JAK/STAT1), *Tnf-α* (dependent on MyD88/NF-κB), and *Il-10* (dependent on IRF3/AKT) mRNA levels. Pretreatment with Dynasore did not affect *Cxcl10* mRNA levels in MIC1-, MIC4-, or LPS-stimulated BMDMs (**Figure 2A**). While *Tnf-α* mRNA levels increased with blockade of endocytosis in MIC-stimulated BMDMs after endocytosis blockage (**Figure 2B**), *Il-10* mRNA levels decreased significantly. Maximal reduction was observed in MIC1-stimulated cells (**Figure 2C**). Upon identify a possible modulation of *Il-10* and *Tnf-α* through the qPCR assay, we also proceeded to the investigation of effect of blocking endocytosis on protein levels of these cytokines released by BMDMs. We confirmed our previous finding that MIC1 and MIC4, similar to LPS, induce increased release of pro-inflammatory TNF-α and anti-inflammatory IL-10 (**Figures 2D, E**, blue bars). Distinct from what was observed in the qPCR results, the pretreatment with Dynasore mildly antagonized MIC induction of TNF-α release (**Figure 2D**, red bars). On the other hand, corroborating with qPCR results, Dynasore treatment effectively quenched IL-10 release by MIC1-, MIC4-, and LPS-stimulated BMDMs (**Figure 2E**, red bars).

We also assayed BMDMs obtained from TLR2-/-, CD14-/-, and TLR4-/- mice. Stimulation of TLR2-/- BMDMs with MICs induced production of TNF-α and IL-10 (**Figures 2F, G**, blue bars) at levels close to those produced by WT BMDMs; in both cell types, levels were typically superior to those released by unstimulated cells (medium). This observation indicates that TLR2 is not critical for microneme protein-induced signaling resulting in TNF-α or IL-10 production. CD14-/- BMDMs produced levels of TNF-α similar to levels produced by WT BMDMs (**Figure 2H**), but reduced levels of IL-10 (**Figure 2I**) in response to MIC1 and MIC4. In the absence of TLR4, BMDMs’ release of IL-10 and TNF-α became unresponsive to MIC exposure (**Figures 2G, H** **)**. Of note, there was a significant production of TNF-α by TLR4-/- and CD14-/- BMDMs in response to LPS (**Figures 2H, J** **)**. The LPS used in this study was not an ultrapure LPS, thus traces of TLR2-agonist might be triggering proinflammatory cytokine release. BMDMs of all assayed phenotypes produced no IL-10 in response to MIC1, MIC4, or LPS when pretreated with Dynasore (**Figures 2E, G, I, K**), while TNF-α levels were mostly preserved.

Taken together, the results presented in this section reveal that TLR4 endocytosis plays a crucial role in MIC1 or MIC4 induced IL-10 production. Absence of TLR4, or blockade of endocytosis in BMDMs completely quenches IL-10 production.

### MIC1 or MIC4 Stimulation Prompts TLR4/CD14-Dependent IRF3 Phosphorylation

LPS stimulation of innate immune cells initiates TLR4 internalization, TRIF activation, and IRF3 phosphorylation. In addition to other effects, IRF3 phosphorylation results in IFN-β and IL-10 secretion (34). Under assayed conditions, we did not verify IFN-β release by BMDMs in response to MICs (**Figure S1**). Because MIC1- or MIC4-stimulated BMDMs vigorously released IL-10 (**Figure 2**), we evaluated whether cell stimulation with MIC1 or MIC4 is implicated in IRF3 phosphorylation.

**Figure 3:**
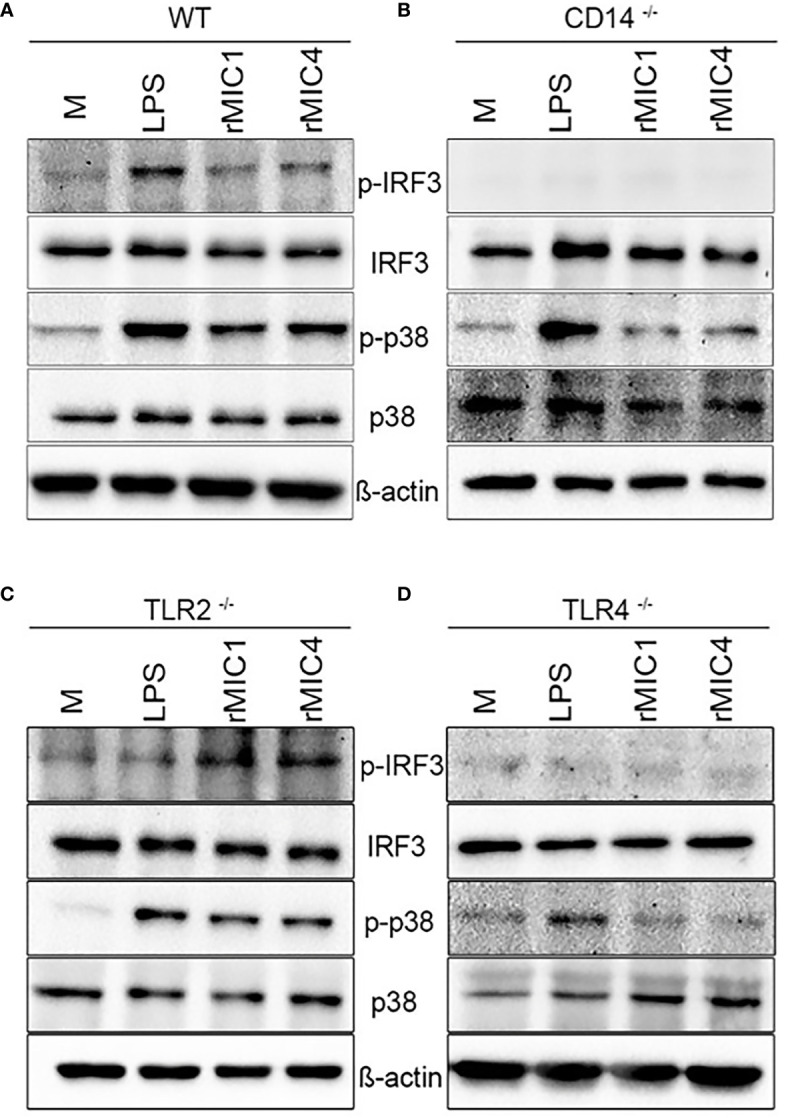
IRF3 phosphorylation in MIC1- or MIC4-stimulated BMDMs depends on CD14 and TLR4. IRF3 and P38 phosphorylation were assessed by western blotting of lysates of WT **(A)**, CD14^-/-^ **(B)**, TLR2^-/-^ **(C)**, and TLR4^-/-^ **(D)** BMDMs, which had been stimulated with medium only (M), LPS, MIC1, or MIC4 for 30 min. Antibodies against phosphorylated and unphosphorylated IRF3 and P38 were used; anti-β-actin was used as a loading control.

As expected, stimulation with MIC1 or MIC4 together with LPS activated p38 phosphorylation independently of the background of the assayed BMDMs, as demonstrated by western blotting (**Figure 3**). This finding is consistent with the high TNF-α concentrations we detected by ELISA (**Figures 2D, F, H, J**) under similar experimental conditions. MIC-induced IRF3 phosphorylation occurred only in WT and TLR2-/- BMDMs (**Figures 3A, C**), similar to our observations regarding IL-10 production (**Figures 2E, G**). Stimulation with MICs did not induce IRF3 phosphorylation or IL-10 production in BMDMs lacking CD14 or TLR4 (**Figures 3B, D**). The shared CD14 and TLR4 dependence of these events reinforces our initial hypothesis of an association between these two events (IL-10 production and IRF3 phosphorylation) (**Figures 2I, K**) in MIC1- or MIC4-stimulated cells. BMDM stimulation with MICs triggers IRF3 phosphorylation in a TLR4-and CD14 dependent manner, as does LPS.

### *Toxoplasma gondii*-Infected BMDMs Produce IL-10 in a TLR4 and Endocytosis-Dependent Manner

MIC1 and MIC4 are carbohydrate-binding proteins released by *T. gondii* in the initial steps of host cell invasion. Herein, we have shown that IL-10 production is an early response of host cells to the contact with MICs, mediated by their interaction with TLR4. To apply our observations to a more realistic infection scenario, we quantified levels of cytokines released by BMDMs when incubated with live parasites instead of with recombinant MIC1 or MIC4.

**Figure 4:**
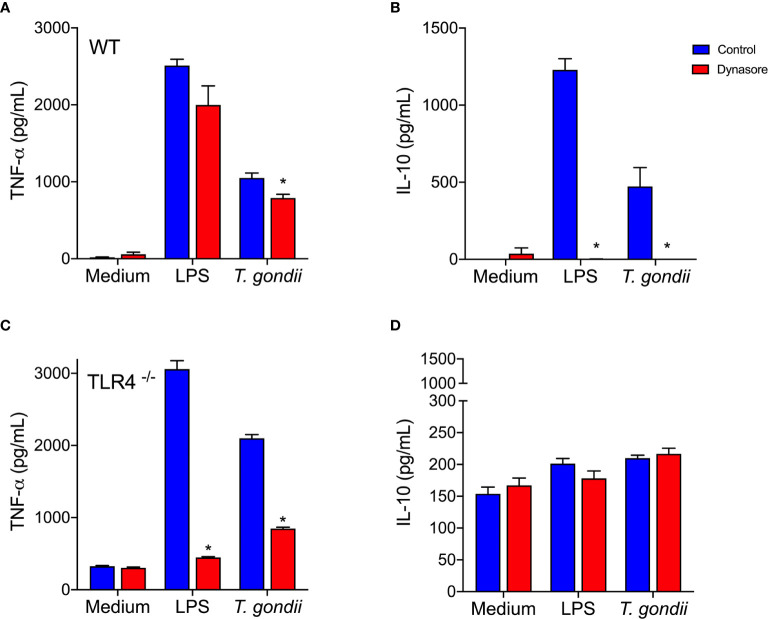
*Toxoplasma gondii*-infected BMDMs produce IL-10 *via* a TLR4 and endocytosis dependent mechanism. WT **(A, B)** and TLR4^-/-^ **(C, D)** BMDMs were pretreated (red bars) or not (control - blue bars) with Dynasore, then stimulated with Medium only or LPS, or infected with *T. gondii* (at a ratio of 3 parasites/BMDM). After 24 h, cell supernatants were analyzed for TNF-α **(A, C)** and IL-10 **(B, D)** levels. Results are expressed as averages + SD of two independent experiments, each performed in triplicate. Two-way ANOVA followed by Tukey’s test was performed (* = p < 0.05 in comparison with control under same stimulation/infection).

Increased TNF-α and IL-10 production followed *in vitro* infection of BMDMs with whole parasites (**Figures 4A, B**, blue bars). Blockade of the BMDMs endocytic pathway slightly decreased TNF-α secretion, but completely abolished parasite induced IL-10 release (**Figures 4A, B**, red bars). When TLR4-/- BMDMs were infected *in vitro* with the parasite, they barely released IL-10 (**Figure 4D**), but they kept producing TNF-α (**Figure 4C**), in levels even greater than the ones detected in WT BMDMs.

Remarkably, the results we obtained using host cells infected *in vitro* with whole living parasites reproduced those obtained by stimulating cells with recombinant MIC1 or MIC4 (**Figures 2D, E**). This indicates that MIC1 and MIC4 contribute to the early IL-10 production observed *in vivo*, which is an essential component of the host defense. Our observations demonstrate the importance of the endocytic pathway, and dependence on TLR4 for IL-10 secretion by *T. gondii* parasitized host cells.

### Endotoxin Tolerance Is Stimulated in BMDMs by MIC1 or MIC4

Exposure of mononuclear phagocytes to LPS induces transient hyporesponsiveness to subsequent LPS stimulus, a state known as endotoxin tolerance (35–37). Its hallmark is the attenuation of TLR4-dependent signaling due to independent mechanisms: TLR4 internalization, and decreased expression of TRIF-dependent genes (38). We found that MIC1 and MIC4 induce IRF3 phosphorylation (**Figure 3**) *via* a cell signaling pathway mediated by TLR4 internalization. To investigate whether MIC mediated TLR4 endocytosis leads to endotoxin tolerance, we first stimulated BMDMs with MIC1, MIC4, or LPS. After 18 h, cells were washed and restimulated for 24 h with LPS.

**Figure 5:**
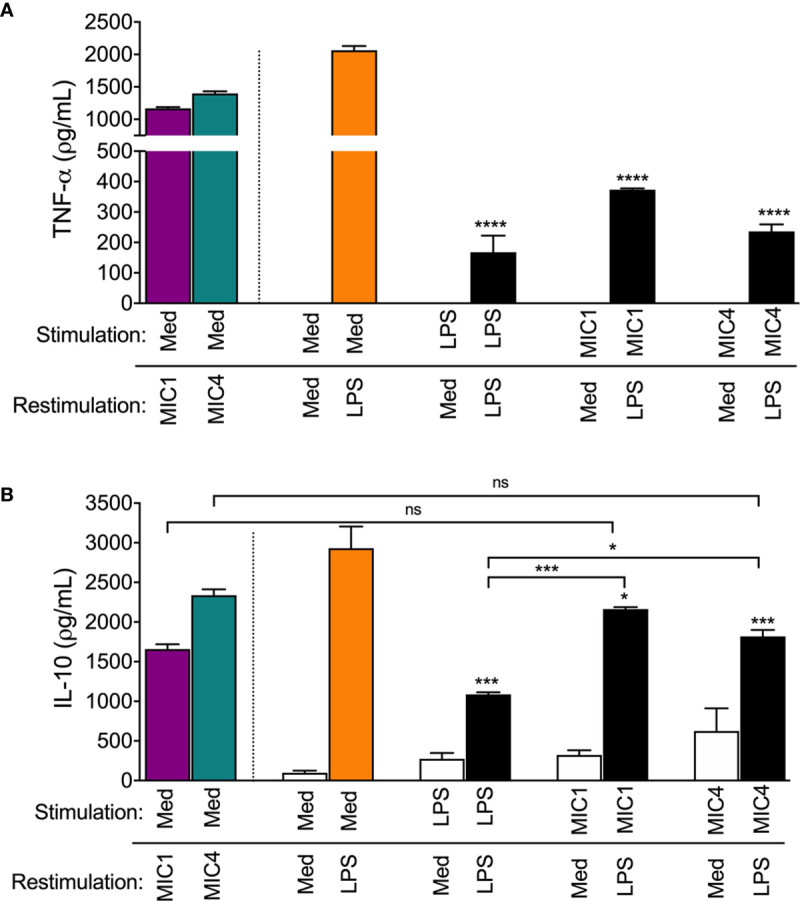
MIC1 and MIC4 induced LPS tolerance in BMDMs. BMDMs were stimulated with Medium only (Med), LPS, MIC1, or MIC4. After 18 h, cells were washed and restimulated for an additional 24 h with medium or LPS. Cell supernatants were assessed for TNF-α **(A)** and IL-10 **(B)** concentrations. Mock-stimulated cells (Med) restimulated with MIC1 or MIC4 (purple and green bars) were used as controls. Results are expressed as averages + SD of three independent experiments, each performed in triplicate. Statistical analysis was performed by two-way ANOVA followed by Tukey’s test. * = p < 0.05, *** = p < 0.001 and **** = p < 0.0001, in comparison with cells stimulated with Med and restimulated with LPS (orange bars), or as indicated.

As expected, we showed that mock-tolerized macrophages (“stimulated” with medium only) produced high concentrations of both TNF-α and IL-10 in response to “restimulation” with MIC1, MIC4, or LPS (**Figures 5A, B**, colored bars compared to Med/Med). We confirmed that in response to LPS restimulation, LPS-stimulated macrophages (LPS/LPS) released 11-fold lower TNF-α levels than mock-tolerized BMDMs (**Figure 5A**, Med/LPS - orange bar). In addition, MIC1- or MIC4-stimulated BMDMs produced 5- and 8-fold lower TNF-α levels, respectively, than mock-tolerized BMDMs when restimulated with LPS (**Figure 5A**). Cells stimulated and restimulated with pairs of MICs (MIC1/MIC1, MIC4/MIC4, MIC1/MIC4, or MIC4/MIC1) allowed us to conclude that homo-and hetero-tolerance to MIC1 and MIC4 have occurred. In these cases, tolerance was manifested by abolishment of TNF-α production, whereas IL-10 levels were mostly preserved (**Table S1**).

**Figure 5B** shows that cells that were restimulated with LPS after initial stimulation with LPS (LPS/LPS), MIC1 (MIC1/LPS), or MIC4 (MIC4/LPS) displayed diminished IL-10 production compared to mock-tolerized macrophages (Med/LPS - orange bar). IL-10 production in response to LPS restimulation was twice as high in macrophages exposed first to MIC1, and 1.5 fold higher in MIC4-exposed macrophages than in LPS-tolerized cells (**Figure 5B**).

Our results suggest that MIC1 and MIC4 induce cell tolerance to endotoxin, manifested by reduced TNF-α production in response to LPS challenge. This observation, together with the high IL-10 release by MIC1- or MIC4-tolerized BMDMs indicates that tolerized cells are rendered, at least temporarily, anti-inflammatory.

## Discussion

This study reports that the *T. gondii* lectins, MIC1 and MIC4, in addition to inducing proinflammatory cytokine release by macrophages, as previously reported (19, 20), stimulate IL-10 production in a TLR4-endocytosis dependent manner. The ability of MIC1 and MIC4 to cause the release of pro-inflammatory cytokines following activation of innate immune cells is attributed to signaling pathways that involve MYD88, TAK-1, and NF-κB nuclear translocation (20). Nonetheless, the mechanisms by which MIC1 and MIC4 induce the anti-inflammatory cytokine IL-10 (19) have not yet been elucidated.

**Figure 6:**
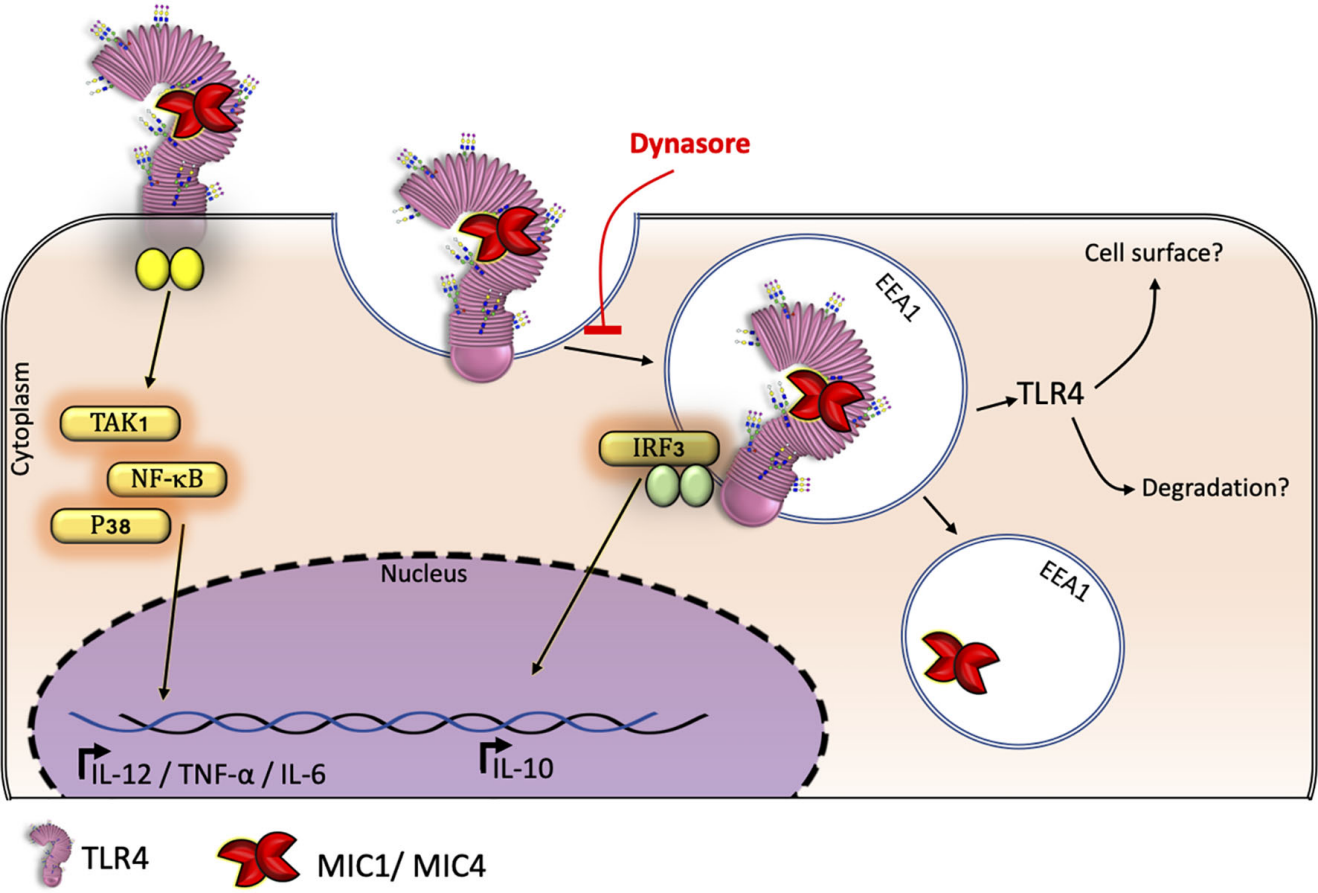
*Tg*MIC1 and *Tg*MIC4 drive TL4 into endosomes inducing BMDM-IL-10 release: a model. BMDMs produce the anti-inflammatory cytokine IL-10 in response to MIC 1 and MIC4 depending on TLR4 internalization from the cell surface. Macrophages subjected to blockage of endocytosis by Dynasore continued to release the proinflammatory cytokine TNF-α but failed to produce IL-10 in response to MIC1 or MIC4 exposure.

Consistent literature indicates that TLR2 agonists induce IL-10 production by antigen-presenting cells (APCs) (39–41). However, we verified that TLR2-deficient BMDMs produce significant IL-10 levels in response to MIC stimulation (**Figure 2G**), indicating that TLR2 is not crucial for induction of IL-10 release. Indeed, the TLR activation-dependent IL-10-release may change in the different immune cell types. Macrophages and myeloid dendritic cells (DCs), but not plasmacytoid DCs, produce IL-10 in response to TLR activation, with macrophages being the higher producers (42). As reviewed by Saraiva and O’Garra (43), optimal IL-10 production induced by LPS, a classical TLR4 agonist, requires activation of both TRIF- and MYD88-dependent pathways (43). By assaying BMDMs that were pretreated with the dynamin inhibitor Dynasore, we showed that MIC1 and MIC4’s ability to induce an anti-inflammatory response requires the integrity of the endocytic pathway. Thus, by interacting with TLR2- and TLR4-associated N-glycans on cell surfaces (19), MIC1 and MIC4 prompt the release of pro-inflammatory cytokines, even by Dynasore-conditioned cells (**Figure 2D**). IL-10 release additionally requires TLR4 endocytosis by MIC1- or MIC4-stimulated macrophages (**Figures 2E** and **6**).

TLR4 endocytosis in response to LPS activates the TRIF-TRAM pathway, leading to IRF3 phosphorylation. This endosomal TLR4 signaling frequently results in IFN-β and CXCL-10 secretion, and negatively regulates inflammation *via* a mechanism referred to as “tolerance” (38). Besides, the up-regulation of type I IFN was shown to enhance the BMDMs release of IL-10 after LPS stimulation (44). Similar to LPS, MIC1 and MIC4 induce TLR4 endocytosis followed by IRF3 phosphorylation (**Figures 1** and **3**). However, their activity diverges from that of LPS in that MIC1 and MIC4 do not cause IFN-β release, as does LPS (**Figure S1**). In addition to IFN-β secretion, IL-10 release was also reported to result from TLR4 endocytosis, *via* a mechanism dependent on the p110δ isoform of the kinase PI(3)K (45). If IL-10 production induced by MIC1 and MIC4 is also dependent in the p110δ isoform of the kinase PI(3)K is still to be investigated. Remarkably, IL-10 secretion induced by LPS, MIC1, and MIC4 is entirely dependent on endocytic pathway integrity, as well as on the presence of TLR4 and CD14 (**Figure 2**).

Although the mechanisms by which MIC1 and MIC4 induce proinflammatory cytokine release have already been explored, questions remain on this issue. For instance, why would *T. gondii* stimulate host cells to mount a potentially lethal response to itself? Indeed, *T. gondii* infections that result in high production of proinflammatory cytokines, including IL-12, TNF, and IFN-γ ultimately control the parasite. Nonetheless, a proinflammatory cytokine storm that would presumably follow *T. gondii* infection does not occur, because it is downregulated by high IL-10 release (46). Therefore, IL-10 is critical for establishing a chronic toxoplasmosis phase, associated with formation of *T. gondii* type II strains (47). Consistently, even when infected with avirulent Type II parasites, IL-10-deficient mice overproduce IFN-γ, TNF-α, and IL-12, leading to exacerbated inflammation, tissue injury, and premature death (29).

To our knowledge, this is the first report describing how *T. gondii* components can induce release of the anti-inflammatory cytokine, IL-10. We therefore highlight two directions in need of further investigation. Firstly, the fact that MIC1- and MIC4-stimulated BMDMs are temporarily LPS-tolerized (**Figure 5**) provides a possible mechanism for evasion of the host inflammatory response by *T. gondii*. Because we already know that MIC1 and MIC4 act on host cells through their carbohydrate recognition domains (CRDs) (19), we hypothesize that during evolution there was a selection for parasites expressing lectin components, favoring host-parasite coexistence. During early infection stage, the balance between inflammatory and anti-inflammatory cytokines produced by the host is controlled, at least partially, by the parasites themselves. During early stages of infection, inflammatory mediators induced by ingested *T. gondii* components activate neutrophil migration, attracting motile parasite reservoirs, whose retrograde transit then spreads *T. gondii* throughout the small intestine (48). Induction of inflammatory mediators thus allows parasites access to different host tissues during early stages of infection. Subsequently, the production of anti-inflammatory mediators can fine-tune host-parasite coexistence during establishment of chronic toxoplasmosis (29, 46, 47). The second aspect to be considered concerns the potential application of recombinant forms of these MICs in immunotherapy against *T. gondii* infection. Administration of either MIC1 or MIC4 confers protection against experimental murine toxoplasmosis, mediated by the Th1 immune response (49). Because MIC1 and MIC4 also induce the anti-inflammatory cytokine IL-10, they are strong candidates for safe vaccines and immunomodulatory agents.

In summary, this study shows that TLR4 but not TLR2 is crucial for IL-10 release induced by the lectins MIC1 and MIC4 from *T. gondii*. Shortly after interacting with TLR4 on BMDMs surface, these lectins are found colocalized with early endosomes. It was also shown that the block of the endocytic pathway strongly impairs the IL-10 secretion while it barely compromises TNF-α release in the cells stimulated with MIC1 and MIC4, suggesting an impairment in endosomal TLR4 signaling pathways, but not the TLR4 signaling triggered on the cell surface. To illustrate the main findings in this study, we propose a graphical model showing the mechanism through which TLR4 endocytosis induced by MIC1 and MIC4 is triggering IRF3 phosphorylation and then IL-10 secretion by BMDMs (**Figure 6**). Lastly, we present evidence suggesting that MIC1 and MIC4, likewise LPS itself, induce BMDMs tolerance to endotoxin.

## Data Availability Statement

The original contributions presented in the study are included in the article/**Supplementary Material**. Further inquiries can be directed to the corresponding author.

## Ethics Statement

The animal study was reviewed and approved by Ethics Committee on Animal Experimentation and Research of the Ribeirão Preto Medical School (FMRP) (protocol number 191/2017).

## Author Contributions

Conceptualization: RR-A, FM-N, MR-B. Experimental design: RR-A, FM-N. Data curation: RR-A, FM-N, AS, JAD. Formal analysis: RR-A, FM-N, AS. Investigation: RR-A, FM-N, AS, JAD. Methodology: RR-A, FM-N, AS, JAD. Project administration: RR-A, FM-N, MR-B. Validation: RR-A, FM-N. Visualization: RR-A, FM-N. Funding acquisition: MR-B. Resources: MR-B. Supervision: MR-B. Writing—original draft. RR-A. Writing—review and editing: RR-A, MR-B. All authors contributed to the article and approved the submitted version.

## Funding

This study was supported by grants from Conselho Nacional de Desenvolvimento Científico e Tecnológico (CNPq - grant 166166/2014 to AS) and Fundação de Amparo à Pesquisa do Estado de São Paulo (FAPESP - grants #2017/02998-0 to RR-A; #2014/13324-1 to FM-N; #2016/14657-0 to JAD; #2018/21708-5, #2016/10446-4, #2013/04088-0 to MR-B; and 2004/08868-0 to Laboratório Multiusuário de Microscopia Confocal – LMMC-FMRP-USP).

## Conflict of Interest

The authors declare that the research was conducted in the absence of any commercial or financial relationships that could be construed as a potential conflict of interest.

## References

[1] Dubey JP. Toxoplasmosis of Animals and Humans. CRC Press (2016). 10.1201/9781420092370.

[2] Montoya JG Liesenfeld O. Toxoplasmosis. The Lancet (2004) 363:1965–76. 10.1016/S0140-6736(04)16412-X 15194258

[3] Hill DE Dubey JP. Toxoplasma gondii. In: Ortega YR Sterling CR, editors. Foodborne Parasites. Cham: Springer International Publishing (2018) p. 119–38. 10.1007/978-3-319-67664-7_6

[4] Wreghitt TG Joynson DHM. Toxoplasma infection in immunosuppressed (HIV-negative) patients. In: Joynson DHM Wreghitt TGE, editors. Toxoplasmosis: A Comprehensive Clinical Guide. Cambridge University Press, Cambridge (2001) p. 178–92. 10.1017/CBO9780511527005.008

[5] Carruthers VB Sibley LD. Sequential protein secretion from three distinct organelles of Toxoplasma gondii accompanies invasion of human fibroblasts. Eur J Cell Biol (1997) 73:114–23. PMC13475723 9208224

[6] Hunter CA Sibley LD. Modulation of innate immunity by Toxoplasma gondii virulence effectors. Nat Rev Microbiol (2012) 10:766–78. 10.1038/nrmicro2858 PMC3689224 23070557

[7] Reiss M Viebig N Brecht S Fourmaux MN Soete M Di Cristina M. Identification and characterization of an escorter for two secretory adhesins in Toxoplasma gondii. J Cell Biol (2001) 153:563–78. 10.1083/jcb.152.3.563 PMC2196004 11157983

[8] Xing M Yang N Jiang N Wang D Sang X Feng Y. A sialic acid-binding protein SABP1 of toxoplasma gondii mediates host cell attachment and invasion. J Infect Dis (2020) 222:126–35. 10.1093/infdis/jiaa072 PMC7296849 32060530

[9] Buzoni-Gatel D Werts C. Toxoplasma gondii and subversion of the immune system. Trends Parasitol (2006) 22(10):448–52. 10.1016/j.pt.2006.08.002 16904378

[10] Yarovinsky F Zhang D Andersen JF Bannenberg GL Serhan CN Hayden MS. Immunology: TLR11 activation of dendritic cells by a protozoan profilin-like protein. Science (80) (2005) 308(5728):1629. 10.1126/science.1109893 15860593

[11] Koblansky AA Jankovic D Oh H Hieny S Sungnak W Mathur R. Recognition of Profilin by Toll-like Receptor 12 Is Critical for Host Resistance to Toxoplasma gondii. Immunity (2013) 11:541–47. 10.1016/j.immuni.2012.09.016 PMC3601573 23246311

[12] Yarovinsky F. Innate immunity to Toxoplasma gondii infection. Nat Rev Immunol (2014) 14:109–21. 10.1038/nri3598 24457485

[13] Debierre-Grockiego F Campos MA Azzouz N Schmidt J Bieker U Resende MG. Activation of TLR2 and TLR4 by Glycosylphosphatidylinositols Derived from Toxoplasma gondii. J Immunol (2007) 179(2):1129–37. 10.4049/jimmunol.179.2.1129 17617606

[14] Kang HK Lee HY Lee YN Jo EJ Kim JI Aosai F. Toxoplasma gondii-derived heat shock protein 70 stimulates the maturation of human monocyte-derived dendritic cells. Biochem Biophys Res Commun (2004) 322(3):899–904. 10.1016/j.bbrc.2004.07.205 15336548

[15] Braun L Brenier-Pinchart MP Yogavel M Curt-Varesano A Curt-Bertini RL Hussain T. A Toxoplasma dense granule protein, GRA24, modulates the early immune response to infection by promoting a direct and sustained host p38 MAPK activation. J Exp Med (2013) 210(10):2071–86. 10.1084/jem.20130103 PMC3782045 24043761

[16] Mercer HL Snyder LM Doherty CM Fox BA Bzik DJ Denkers EY. Toxoplasma gondii dense granule protein GRA24 drives MyD88-independent p38 MAPK activation, IL-12 production and induction of protective immunity. PloS Pathog (2020). 10.1371/journal.ppat.1008572 PMC7255617 32413093

[17] Lourenço EV Pereira SR Faça VM Coelho-Castelo AAM Mineo JR Roque-Barreira MC. Toxoplasma gondii micronemal protein MIC1 is a lactose-binding lectin. Glycobiology (2001) 11:541–7. 10.1093/glycob/11.7.541 11447133

[18] Lourenço EV Bernardes ES Silva NM Mineo JR Panunto-Castelo A Roque-Barreira MC. Immunization with MIC1 and MIC4 induces protective immunity against Toxoplasma gondii. Microbes Infect (2006) 8:1244–51. 10.1016/j.micinf.2005.11.013 16616574

[19] Sardinha-Silva A Mendonça-Natividade FC Pinzan CF Lopes CD Costa DL Jacot D. The lectin-specific activity of Toxoplasma gondii microneme proteins 1 and 4 binds Toll-like receptor 2 and 4 N-glycans to regulate innate immune priming. PloS Pathog (2019) 15:e1007871. 10.1371/journal.ppat.1007871 31226171 PMC6608980

[20] Mendonça-Natividade FC Lopes CD Ricci-Azevedo R Sardinha-Silva A Pinzan CF Alegre-Maller ACP. Receptor heterodimerization and co-receptor engagement in TLR2 activation induced by MIC1 and MIC4 from Toxoplasma gondii. Int J Mol Sci (2019) 20:5001. 10.3390/ijms20205001 PMC6829480 31658592

[21] Paing MM Tolia NH. Multimeric Assembly of Host-Pathogen Adhesion Complexes Involved in Apicomplexan Invasion. PloS Pathog (2014) 10:e1004120. 10.1371/journal.ppat.1004120 PMC4055764 24945143

[22] Brecht S Carruthers VB Ferguson DJP Giddings OK Wang G Jakle U. The Toxoplasma Micronemal Protein MIC4 Is an Adhesin Composed of Six Conserved Apple Domains. J Biol Chem (2001) 276:4119–27. 10.1074/jbc.M008294200 11053441

[23] Marchant J Cowper B Liu Y Lai L Pinzan C Marq JB. Galactose recognition by the apicomplexan parasite Toxoplasma gondii. J Biol Chem (2012) 287:16720–33. 10.1074/jbc.M111.325928 PMC3351351 22399295

[24] Ricci-Azevedo R Roque-Barreira M-C Gay NJ. Targeting and recognition of toll-like receptors by plant and pathogen lectins. Front Immunol (2017) 8:1820. 10.3389/fimmu.2017.01820 29326706 PMC5741612

[25] Suzuki Y Orellana MA Schreiber RD Remington JS. Interferon-γ: The major mediator of resistance against Toxoplasma gondii. Science (80) (1988) 240:516–8. 10.1126/science.3128869 3128869

[26] Yarovinsky F. Toll-like receptors and their role in host resistance to Toxoplasma gondii. Immunol Lett (2008) 119(1–2):17–21. 10.1016/j.imlet.2008.05.007 18617274

[27] Jankovic D Kugler DG Sher A. IL-10 production by CD4+ effector T cells: A mechanism for self-regulation. Mucosal Immunol (2010) 3:239–46. 10.1038/mi.2010.8 PMC4105209 20200511

[28] Couper KN Blount DG Riley EM. IL-10: The Master Regulator of Immunity to Infection. J Immunol (2008) 180:5771–7. 10.4049/jimmunol.180.9.5771 18424693

[29] Gazzinelli RT Wysocka M Hieny S Scharton-Kersten T Cheever A Kühn R. In the absence of endogenous IL-10, mice acutely infected with Toxoplasma gondii succumb to a lethal immune response dependent on CD4+ T cells and accompanied by overproduction of IL-12, IFN-gamma and TNF-alpha. J Immunol (1996) 157:798–805. 8752931

[30] Costa Mendonça-Natividade F Ricci-Azevedo R de Oliveira Thomaz SM Roque-Barreira MC. Production and characterization of MIC1: A lectin from toxoplasma gondii. In: Hirabayashi J. (ed) LecQn PurificaQon and Analysis. Methods in Molecular Biology. New York: Humana (2020) 2132. 10.1007/978-1-0716-0430-4_38 32306346

[31] Khan A Grigg ME. Toxoplasma gondii: Laboratory maintenance and growth. Curr Protoc Microbiol (2017) 44:20c.1.1. 10.1002/cpmc.26 PMC5537724 28166387

[32] Marim FM Silveira TN Lima DS Jr Zamboni DS. A Method for Generation of Bone Marrow-Derived Macrophages from Cryopreserved Mouse Bone Marrow Cells. PloS One (2010) 5:e15263. 10.1371/journal.pone.0015263 21179419 PMC3003694

[33] Schindelin J Arganda-Carreras I Frise E Kaynig V Longair M Pietzsch T. Fiji: An open-source platform for biological-image analysis. Nat Methods (2012) 9:672–82. 10.1038/nmeth.2019 PMC3855844 22743772

[34] Li J Lee DSW Madrenas J. Evolving Bacterial Envelopes and Plasticity of TLR2-Dependent Responses: Basic Research and Translational Opportunities. Front Immunol (2013). 10.3389/fimmu.2013.00347 PMC3808894 24191155

[35] Henricson BE Benjamin WR Vogel SN. Differential cytokine induction by doses of lipopolysaccharide and monophosphoryl lipid A that result in equivalent early endotoxin tolerance. Infect Immun (1990). 10.1128/iai.58.8.2429-2437.1990 PMC258837 1695201

[36] Rajaiah R Perkins DJ Polumuri SK Zhao A Keegan AD Vogel SN. Dissociation of Endotoxin Tolerance and Differentiation of Alternatively Activated Macrophages. J Immunol (2013) 190(9):4763–72. 10.4049/jimmunol.1202407 PMC3633613 23543762

[37] Foster SL Hargreaves DC Medzhitov R. Gene-specific control of inflammation by TLR-induced chromatin modifications. Nature (2007) 447:972–8. 10.1038/nature05836 17538624

[38] Rajaiah R Perkins DJ Ireland DDC Vogel SN Kagan JC. CD14 dependence of TLR4 endocytosis and TRIF signaling displays ligand specificity and is dissociable in endotoxin tolerance. Proc Natl Acad Sci USA (2015) 112(27):8391–6. 10.1073/pnas.1424980112 PMC4500272 26106158

[39] Netea MG Sutmuller R Hermann C Van der Graaf CAA Van der Meer JWM van Krieken JH. Toll-Like Receptor 2 Suppresses Immunity against Candida albicans through Induction of IL-10 and Regulatory T Cells. J Immunol (2004) 172(6):3712–8. 10.4049/jimmunol.172.6.3712 15004175

[40] Dillon S Agrawal A Van Dyke T Landreth G McCauley L Koh A. A Toll-Like Receptor 2 Ligand Stimulates Th2 Responses In Vivo, via Induction of Extracellular Signal-Regulated Kinase Mitogen-Activated Protein Kinase and c-Fos in Dendritic Cells. J Immunol (2004) 172(8):4733–43. 10.4049/jimmunol.172.8.4733 15067049

[41] Hu X Paik PK Chen J Yarilina A Kockeritz L Lu TT. IFN-γ Suppresses IL-10 Production and Synergizes with TLR2 by Regulating GSK3 and CREB/AP-1 Proteins. Immunity (2006) 24(5):563–74. 10.1016/j.immuni.2006.02.014 16713974

[42] Boonstra A Rajsbaum R Holman M Marques R Asselin-Paturel C Pereira JP. Macrophages and Myeloid Dendritic Cells, but Not Plasmacytoid Dendritic Cells, Produce IL-10 in Response to MyD88- and TRIF-Dependent TLR Signals, and TLR-Independent Signals. J Immunol (2006) 177(11):7551–8. 10.4049/jimmunol.177.11.7551 17114424

[43] Saraiva M O’Garra A. The regulation of IL-10 production by immune cells. Nat Rev Immunol (2010) 10:170–81. 10.1038/nri2711 20154735

[44] Chang EY Guo B Doyle SE Cheng G. Cutting Edge: Involvement of the Type I IFN Production and Signaling Pathway in Lipopolysaccharide-Induced IL-10 Production. J Immunol (2007) 178(11):6705–9. 10.4049/jimmunol.178.11.6705 17513714

[45] Aksoy E Taboubi S Torres D Delbauve S Hachani A Whitehead MA. The p110δ isoform of the kinase PI(3)K controls the subcellular compartmentalization of TLR4 signaling and protects from endotoxic shock. Nat Immunol (2012) 13:1045–54. 10.1038/ni.2426 PMC4018573 23023391

[46] Wilson EH Wille-Reece U Dzierszinski F Hunter CA. A critical role for IL-10 in limiting inflammation during toxoplasmic encephalitis. J Neuroimmunol (2005) 165(1–2):63–74. 10.1016/j.jneuroim.2005.04.018 16005735

[47] Jeong YI Hong SH Cho SH Park MY Lee SE. Induction of IL-10-producing regulatory B cells following Toxoplasma gondii infection is important to the cyst formation. Biochem Biophys Rep (2016) 7:91–7. 10.1016/j.bbrep.2016.05.008 PMC5613251 28955894

[48] Coombes JL Charsar BA Han SJ Halkias J Chan SW Koshy AA. Motile invaded neutrophils in the small intestine of Toxoplasma gondii-infected mice reveal a potential mechanism for parasite spread. Proc Natl Acad Sci USA (2013) 10:E1913–22. 10.1073/pnas.1220272110 PMC3666704 23650399

[49] Pinzan CF Sardinha-Silva A Almeida F Lai L Lopes CD Lourenço EV. Vaccination with recombinant microneme proteins confers protection against experimental toxoplasmosis in mice. PloS One (2015) 10:e0143087. 10.1371/journal.pone.0143087 26575028 PMC4648487

