# Supplementary material for: Microneme Proteins 1 and 4 From Toxoplasma gondii Induce IL-10 Production by Macrophages Through TLR4 Endocytosis
Source: Front Immunol. 2021 Apr 12;12:655371. doi: 10.3389/fimmu.2021.655371 (PMC8071938; doi:10.3389/fimmu.2021.655371)
Supplement: Supplementary file 1 [file DataSheet_1.pdf]

Supplementary Figure 1

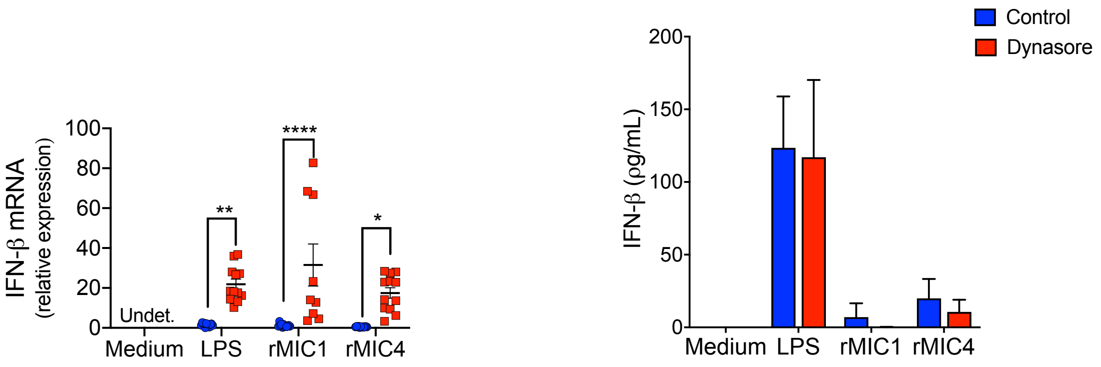

Supplementary Figure 1: MIC1 and MIC4 do not stimulate IFN-β production. WT BMDMs were pretreated (red squares/bars) or not (control - blue circles/bars) with Dynasore, then stimulated with Medium only, LPS, MIC1, or MIC4 for 5 h (**A**) or 24 h (**B**). Extracted RNA was reverse transcribed into cDNA, and *Ifn-β* expression was analyzed by real-time PCR (**A**). Relative expression was determined as described in “Materials and Methods”. Results obtained from Dynasore treated cells were compared to results from control cells stimulated the same way. Results are expressed as averages ± SD of four experiments, each performed in triplicate. Statistical analysis was performed by two-way ANOVA followed by Tukey's test (* = $p < 0.05$, ** = $p < 0.01$, **** = $p < 0.0001$). Cell supernatants were analyzed by ELISA for IFN-β concentrations (**B**). Results are expressed as averages ± SD of two independent experiments, each performed in triplicate.

Supplementary Table 1

Supplementary table 1 – Endotoxin tolerance assay. Levels TNF-α and IL-10 secreted by BMDMs 18h-stimulated by Medium only, LPS, MIC1 and MIC4 and 24h-restimulated, after washing, with the same stimuli panel.

Stimulation	Restimulation	Cytokine levels (average ± SD - *p*g/mL)	
		**TNF-α**	**IL-10**
Med	Med	ND	99,15±43,63
	LPS	2063,78±113,83	2930,99±476,39
	MIC1	1168,26±34,41	1658,82±105,58
	MIC4	1398,10±60,10	2337,96±130,39
LPS	Med	ND	274,37±125,82
	LPS	167,63±94,67	1087,53±43,63
	MIC1	51,31±13,52	729,62±49,28
	MIC4	321,79±188,70	840,43±118,04
MIC1	Med	ND	323,78±100,19
	LPS	373,64±7,28	2164,25±39,93
	MIC1	19,08±8,75	1175,88±77,94
	MIC4	59,72±27,36	1971,07±837,83
MIC4	Med	ND	624,79±495,21
	LPS	236,30±39,96	1818,32±140,71
	MIC1	10,85±10,29	1017,14±60,66
	MIC4	12,25±12,34	1043,23±69,64

ND stands for not detected.
